# Macropinosomes as units of signal transduction

**DOI:** 10.1098/rstb.2018.0157

**Published:** 2018-12-17

**Authors:** Joel A. Swanson, Sei Yoshida

**Affiliations:** Department of Microbiology and Immunology, University of Michigan Medical School, Ann Arbor, MI 48109-5620, USA

**Keywords:** growth factors, actin, phosphatidylinositol 3-kinase

## Abstract

Macropinosome formation occurs as a localized sequence of biochemical activities and associated morphological changes, which may be considered a form of signal transduction leading to the construction of an organelle. Macropinocytosis may also convey information about the availability of extracellular nutrients to intracellular regulators of metabolism. Consistent with this idea, activation of the metabolic regulator mechanistic target of rapamycin complex-1 (mTORC1) in response to acute stimulation by growth factors and extracellular amino acids requires internalization of amino acids by macropinocytosis. This suggests that macropinocytosis is necessary for mTORC1-dependent growth of metazoan cells, both as a route for delivery of amino acids to sensors associated with lysosomes and as a platform for growth factor-dependent signalling to mTORC1 via phosphatidylinositol 3-kinase (PI3K) and the Akt pathway. Because the biochemical signals required for the construction of macropinosomes are also required for cell growth, and inhibition of macropinocytosis inhibits growth factor signalling to mTORC1, we propose that signalling by growth factor receptors is organized into stochastic, structure-dependent cascades of chemical reactions that both build a macropinosome and stimulate mTORC1. More generally, as discrete units of signal transduction, macropinosomes may be subject to feedback regulation by metabolism and cell dimensions.

This article is part of the Theo Murphy meeting issue ‘Macropinocytosis’.

## 1. Introduction

**Figure 1.**
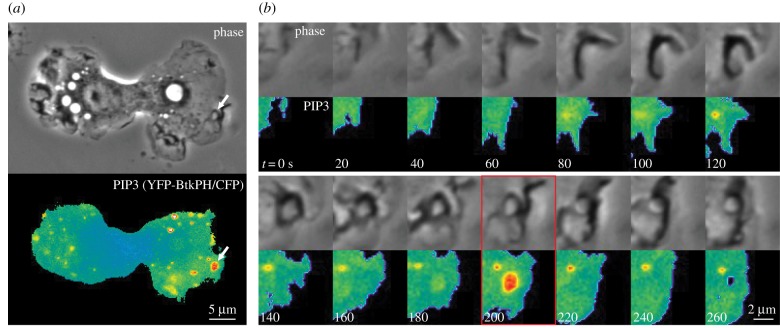
Transient appearance of phosphatidylinositol (3,4,5)-trisphosphate (PIP_3_) in macropinocytic cups. Phase-contrast (phase) and ratiometric fluorescence images (PIP3) of a bone-marrow-derived macrophage expressing YFP-BtkPH and CFP and stimulated with macrophage colony-stimulating factor (M-CSF). (*a*) Entire cell. (*b*) Time sequence of the region of the cell indicated by the arrows in (*a*) (see also electronic supplementary material, Video S1). Indicated times are relative to the beginning of ruffle formation. Increased PIP_3_ concentrations (i.e. increased YFP-chimera/CFP ratios) are indicated by warm colours. PIP_3_ concentrations increased inside macropinocytic cups at 180 and 200 s and returned to baseline at 220 s. Scale bars: (*a*) 5 µm, (*b*) 2 µm. Methods described in Yoshida *et al.* [2].

**Figure 2.**
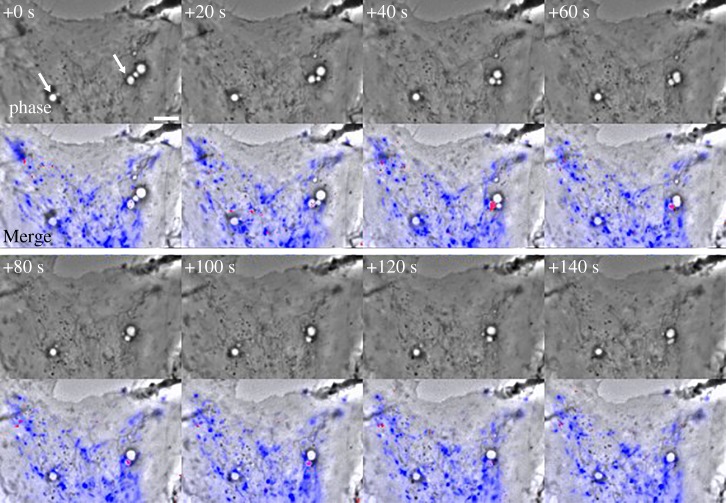
Solute size-dependent delivery of dye from endolysosomes into macropinosomes by piranhalysis. Time-series of a macrophage stimulated with M-CSF after pre-loading endolysosomes with Lucifer yellow (LY) and Texas Red-labelled dextran (TRDx) (see also electronic supplementary material, Video S2). One image set was collected every 20 s. Top panels: Phase-contrast. Indicated times, in seconds, are relative to the first frame. Arrows at +0 s indicate two macropinosomes which received LY and TRDx during the sequence. Bottom panels: phase-contrast with a blue overlay indicating endolysosomes containing both LY and TRDx fluorescence and red overlay indicating macropinosomes containing increased concentrations of LY relative to TRDx. Preferential labelling of macropinosomes with LY indicated the molecular size-selective transfer of fluid-phase probes between the interacting organelles (i.e. LY entered macropinosomes from endolysosomes earlier than did TRDx). Scale bar: 5 µm. Adapted from a supplementary movie described in Yoshida *et al.* [6].

Macropinosomes are transient endocytic organelles which form spontaneously or in response to chemical or physical stimulation. Although the details of macropinosome formation vary among different cell types [1], the process always involves cell surface protrusions that enclose extracellular fluids into plasma membrane-derived intracellular vesicles, which may range in diameter from 0.2 µm to larger than 8 µm (macropinosomes are distinguished from micropinosomes, which are smaller than 0.2 µm, the limit of resolution for light microscopy). In macrophages and fibroblasts, macropinosomes originate from actin-rich, cell surface ruffles that reorganize into cups or circular dorsal ruffles, then close at their distal margins and separate into the cytoplasm from the plasma membrane as vacuoles. The profiles of these nascent macropinosomes are often irregularly shaped, but mature within seconds into rounded, swollen-looking vacuoles. Rounded macropinosomes frequently fuse with other macropinosomes, migrate centripetally from the cell periphery, and shrink in size (figure 1*a*; electronic supplementary material, Video S1). In macrophages, macropinosomes mature by acquiring markers of early endosomes then late endosomes before fusing with lysosomes, all within 15 min of their formation [3]. They may form intravacuolar vesicle inclusions reminiscent of multivesicular bodies [4]. Fusion between macropinosomes and lysosomes occurs by direct and complete fusion, or by transient association and dissociation of macropinosomes and lysosomes, a process called ‘pyranhalysis’ [5] or ‘kiss-and-run’ [6]. Such transient interactions can lead to molecular size-dependent, differential delivery of solute contents between macropinosomes and lysosomes (figure 2; electronic supplementary material, Video S2) [6,7]. In some cells, macropinosomes recycle directly to the cell surface without fusing with lysosomes [8].

Compared to smaller clathrin-coated vesicles (0.15 µm diameter), macropinosomes internalize relatively large quantities of plasma membrane, water and solutes [9]. Cells can maintain high rates of macropinocytosis for hours by returning membrane to the cell surface via small recycling vesicles with accompanying water flux across membranes and out of the cell [10]. Internalized solutes concentrate into lysosomes, where they may accumulate or be degraded by acid hydrolases. Thus, because of their large size and mechanism of maturation, macropinosomes can be considered as mechanical pumps that move water through cytoplasm and efficiently deliver extracellular solutes into lysosomes [7]. This efficient delivery of extracellular solutes into lysosomes may explain how, in actively macropinocytic cells, cell growth can be supported by the ingestion of extracellular proteins [11–14].

In conventional models of signal transduction, an extracellular stimulus initiates changes in intracellular chemistry, which lead to alterations of cell behaviour or metabolism. Typically, ligand binding to cell surface receptors initiates chemical reactions that modify phospholipid chemistry and protein function, and those modifications reach intracellular targets by diffusion or membrane traffic. The cellular response may be localized to a region of the surface, as, for example, during phagocytosis; it may occur throughout the cell, as in metabolic regulation; or it may be graded along the length of the cell, as in chemotactic migration [15]. Intracellular signals can also originate independently of cell surface receptors, leading to self-organized patterns of cellular motility responses [15,16].

Macropinosomes can form in response to receptor-mediated signals or as self-organized structures formed independently of external stimuli [17,18]. Whether macropinocytosis is spontaneous or induced, the construction of the organelle requires a sequence of intracellular signals that organize the sequence of different movements: actin polymerization for ruffle protrusion followed by contractile activities that close the organelle into the cell. The signals associated with macropinocytosis may be strictly limited to organizing the construction and disposal of this transient organelle. However, they may also influence cell function by conveying additional information. For example, as a mechanism for internalizing large portions of plasma membrane, macropinocytosis could regulate levels of cell surface receptors or transport proteins. Accordingly, clathrin-independent endocytosis, of which macropinocytosis is a subset, can internalize plasma membrane proteins that are not cleared by concentration into clathrin-coated vesicles [19].

Here we consider the roles for signalling molecules in the formation and maturation of macropinosomes and the evidence that macropinosomes convey signals that modulate cell metabolism and growth. We consider two kinds of signal: (i) those chemical activities which organize membranes and the cytoskeleton for macropinosome formation and trafficking and (ii) activities associated with macropinocytosis which have separate functions related to metabolism. We propose that macropinosomes serve as discrete, independent units of signalling for cell growth, whose magnitude may be modulated by feedback stimulation or inhibition.

## 2. Localized signals essential to macropinosome formation and maturation

Macropinosome formation exploits biochemical pathways associated with many kinds of receptor, including growth factor receptors, chemoattractant receptors and Fc receptors [18,20]. Upon binding their cognate ligands, these receptors recruit from cytoplasm various adapter proteins and enzymes, including tyrosine kinases, phospholipase C-γ1 (PLCγ1) and isoforms of class I phosphatidylinositol 3-kinase (PI3K), which lead to various cellular responses. PI3K synthesizes phosphatidylinositol (3,4,5)-trisphosphate (PIP_3_) from phosphatidylinositol (4,5)-bisphosphate (PI(4,5)P_2_). In non-transformed cells, plasma membrane concentrations of PIP_3_ are maintained low by the activity of the 3′-phosphatase PTEN [21] (Many kinds of cancer cell have elevated levels of PIP_3_ due to activating mutations of PI3K or to inactivating mutations of PTEN [22].) Elevated concentrations of PIP_3_ in membranes activate proteins with PIP_3_-binding domains, including Akt, PLCγ1, 3′-phosphoinositide-dependent protein kinase-1 (PDK1) [23], and guanine-nucleotide exchange factors (GEFs) that activate the small GTPases Rac1 and Ras [24,25]. These signalling proteins figure prominently in signalling associated with plasma membrane receptors.

PI3K is required for most forms of macropinocytosis. In some cells, inhibition of PI3K inhibits ruffling, likely through inhibition of PIP_3_-activated Rac1 GEFs [26]. In most cells, however, general PI3K inhibitors do not reduce ruffling in response to the growth factors, but rather inhibit a contractile activity that closes macropinocytic cups into macropinosomes [27,28]. Fluorescent reporters of PIP_3_ expressed in living cells reveal PIP_3_-rich membrane within macropinocytic cups [2,29] (figure 1*b*). The increased concentration of PIP_3_ in cups is transient and precedes cup closure, consistent with a role for a PI3K-dependent activity in the closure process [2].

Other activities associated with receptor signalling are necessary for macropinosome formation. Rac1, a GTPase that regulates the actin cytoskeleton, and its effectors p21-activated kinase-1 (Pak1) and Ctb1/BARS are required for macropinocytosis [30,31]. Rac1 is transiently activated in macropinocytic cups, coincident with the PIP_3_ spike [2]. Rac1 supports ruffling and cup formation, but must be converted to its inactive GDP-bound form for cup closure to occur [32]. PLCγ1, which hydrolyses PI(4,5)P_2_ to inositol trisphosphate and diacylglycerol (DAG), is required for macropinosome formation [33,34]. DAG activates protein kinase C (PKC), which directs cup closure by an unknown mechanism. PLCγ contains a PIP_3_-binding domain required for membrane binding and activity [35]; and PLCγ1 may be activated by the elevated concentrations of PIP_3_ in cups. Macropinocytosis occurs independently of class I PI3K in macrophages stimulated by phorbol myristate acetate (PMA) [34], which is a DAG mimetic. The PI3K-independence of PMA-stimulated macropinocytosis suggests that the DAG-dependent activities of macropinosome closure function downstream of PI3K in the signalling pathway. This sequence is supported by fluorescence microscopic studies localizing both PIP_3_ (Btk-PH) and DAG (C1δ-PH) in single cells [34]. PMA-stimulated macropinocytosis is sensitive to PI3K inhibitors with broader specificity, which suggests a role for other classes of PI3K acting downstream of DAG and PKC. Other molecules necessary for macropinosome formation and other kinds of signal transduction are DAG kinase [36], Rab5a [37], Cdc42 [38], Arf6 [39], RhoG [40], Trio [41] and phospholipase D [42]. Because signalling for macropinocytosis has been analysed thoroughly in only a few cell types, it remains possible that the contributions of various signalling molecules to macropinosome formation may be different in other cells or in different kinds of macropinocytic response (e.g. constitutive versus induced).

### (a) Signal cascades are restricted to macropinocytic cups

**Figure 3.**
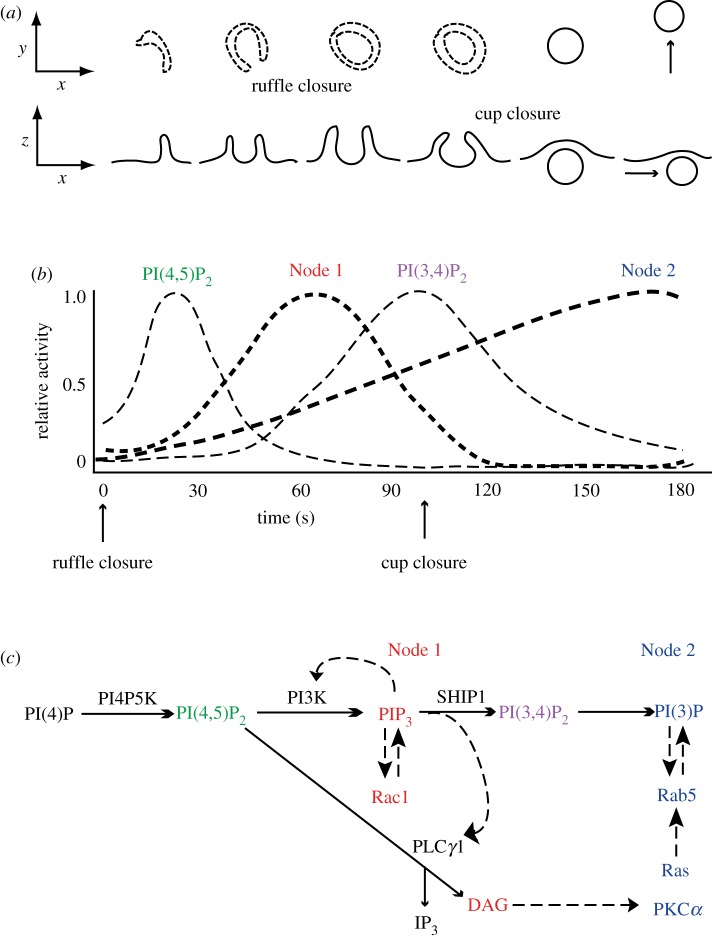
Signal cascades within circular ruffles and macropinosomes. (*a*) Diagram of the stages of macropinosome formation. The top row shows a side view and the bottom row shows a top view (as observed in the microscope) of plasma membrane and macropinosome membranes. Ruffle closure is the formation of a complete circular ruffle, comprised entirely of plasma membrane. Cup closure occurs when the macropinosome separates from the plasma membrane. (*b*) The timing of signals in cups relative to ruffle closure and cup closure. The sequence of chemical transformations occurring between ruffle closure and cup closure begins with a transient increase in PI(4,5)P_2_, followed by increases of PIP_3_, DAG and the activity of Rac1 (Node 1), and later by a transient increase of PI(3,4)P_2_. Within Node 1, the increase in DAG follows shortly after the PIP_3_ spike [34]. The levels of PI(3)P and the activities of Ras, Rab5 and PKC*α* (Node 2) increase continuously in the cup, peaking after cup closure and the transient increases of PI(4,5)P_2_, PIP_3_, DAG and PI(3,4)P_2_. (*c*) Proposed sequence of chemical changes in cups and macropinosomes. Solid lines with small arrowheads indicate precursor–product relationships. Hatched lines with large arrowheads indicate activation pathways enhanced inside circular ruffles. Red font indicates signals comprising Node 1; blue font indicates signals comprising Node 2. Adapted from Yoshida *et al.* and Welliver *et al.* [34,43].

Macrophages stimulated acutely with macrophage colony-stimulating factor (M-CSF) show a striking asynchrony in the timing of the signals that appear at their surfaces. Rac1 activity and PIP_3_ concentrations increase initially at cell margins, but then settle into a sustained pattern of transient spikes of activity, localized to macropinocytic cups and appearing at different times after stimulation [2] (figure 1*b*; electronic supplementary material, Video S1). Moreover, a full sequence of biochemical responses to M-CSF unfolds during the formation of each macropinosome. These responses include transient increases in PI(4,5)P_2_, DAG, PI(3,4)P_2_, PI3P and recruitment of Rab5a, Rab20, Rab21, Ras and PKC*α* [2,43,44] (figure 3). Maekawa *et al*. [45] described a similar sequence of activities required for macropinocytosis in *Caenorhabditis elegans* cells and identified essential roles for phosphoinositide phosphatases MTMR6 and INPP4P, and a Ca^++^-activated K^+^ channel (KCa3.1) in macropinosome formation. In macrophages, the irregular timing of these signal cascades relative to when M-CSF was added, and the correlation of the signal cascades with closure of ruffles into cups, indicate that signal propagation after receptor activation is contingent on the formation of cup domains in plasma membrane. Thus, the signals used to build macropinosomes are confined to cups and the closing macropinosome.

### (b) Cup domains of plasma membrane are isolated from the contiguous membrane outside of the cups

How can signal cascades be limited to the macropinocytic cups when the membrane comprising those very cups is still part of the plasma membrane? One possible explanation is that the structure of the cup forms a barrier that confines lateral diffusion of membrane proteins or lipids. In macrophages expressing photoactivatable green fluorescent protein (GFP) tethered to the inner leaflet of plasma membrane, cup-shaped ruffles inhibited the lateral diffusion of GFP into or out of those cups [46]. This suggests that the circular barrier of the cup facilitates signal propagation, perhaps through feedback amplification of PI3K or other signal molecules.

However, physical barriers may not be required for the construction of large subdomains of plasma membrane. Axenic strains of the soil ameba *Dictyostelium discoideum* exhibit increased macropinocytosis due to mutations in the Ras GAP neurofibromin, which allows them to grow in liquid medium [11]. Macropinosome formation in these cells is organized by PI3K and Ras, whose activities are confined to large patches of plasma membrane surrounded by a narrow rim of the actin-regulatory protein SCAR/WAVE at the advancing edges of macropinocytic cups. Veltman *et al*., [16] showed that PIP_3_/Ras patch formation in *D. discoideum* occurred in cells whose actin cytoskeleton was disrupted by latrunculin A/B, which suggests that this large domain of plasma membrane does not require an actin-based diffusion barrier to maintain lateral heterogeneity. Other possible mechanisms of restricting lateral diffusion of plasma membrane molecules have been described [47]. At present, however, the physical properties and molecular components of cups essential for maintaining the sharp boundary of cup domains (PIP_3_ spikes and Ras patches) remain largely undefined.

### (c) Signalling for macropinocytosis is similar to signalling for phagocytosis

Ingestion of particles by phagocytosis entails activities of the actin cytoskeleton which are much like the movements of macropinocytosis. In Fc receptor-mediated phagocytosis by macrophages, PI3K is required for ingestion of particles larger than 3 µm diameter [27,48]. Inhibition of PI3K does not prevent phagocytic cup formation, but rather inhibits a contractile activity that closes the cup into a phagosome [27]. Fluorescence microscopic methods showed PIP_3_ and active Rac1 localizing to phagocytic cups throughout the ingestion process [49]. Förster resonance energy transfer-based microscopy of other small GTPases showed two distinct kinds of cytoskeletal regulation associated with phagosome formation: active Cdc42 and Arf6 localized to early stages of cup formation and to distal regions of cups, whereas active Rac2 and Arf1 appeared later and localized to the proximal, basal regions of cups [49]. This spatially organized conversion of one category of signals to another (Rac1/Cdc42/Arf6 to Rac1/Rac2/Arf1) within phagocytic cups was named the ‘signal transition’ [50].

The signal transition during phagocytosis is regulated by PI3K. Inhibition of PI3K does not limit early signals or cup initiation, but blocks the appearance of the late signals, indicating that PI3K is required for the transition from early to late signals. The signal transition during phagocytosis may be regulated by a PIP_3_ concentration threshold. Macrophages fed particles with different surface densities of opsonizing IgG showed all-or-none ingestion responses. Over a wide range of IgG densities on particle surfaces, all particles stimulated actin-dependent movements over the particle surface and generation of PIP_3_ in the plasma membrane near the particle, but the ingestion of particles with low IgG densities stalled [51]. Particles with higher densities of IgG were ingested completely, generated more PIP_3_ in phagocytic cups, and recruited the late stage protein PKC*ε* as the cups closed into phagosomes. These studies indicated that a threshold concentration of PIP_3_ in phagosomal membranes must be exceeded to allow completion of phagocytosis. The PIP_3_ concentration threshold could regulate the signal transition from early activities (actin polymerization and cup extension) to late activities (cup contraction and closure).

In macropinocytosis as in phagocytosis, distinct activities must be organized spatially on a relatively large scale, first to assemble the cup, then to close the cup into a macropinosome. Like phagocytosis, macropinosome formation may be modulated by the magnitude of PI3K signalling, in determining either the size of the macropinosome or the ability of cups to close into macropinosomes. Ruffling is a kind of exploratory behaviour, in which actin-rich protrusions extend and withdraw into the cell, and only occasionally close into macropinosomes. This behaviour is regulated by the readily reversible chemistry of PIP_3_ synthesis (PI3K versus PTEN), similar to the excitable signal transduction networks associated with chemotaxis [15]. By contrast, the later stages of macropinosome formation require the hydrolysis of PI(4,5)P_2_ to DAG, which may, by its irreversible nature, signal an all-or-none commitment to completing macropinosome closure. Thus, a cell's ability to make a macropinosome may be modulated by feedback that affects its ability to create a patch of the membrane with sufficiently high concentrations of PIP_3_ to trigger the late signals necessary for cup closure.

### (d) Downstream effectors of PI3K may be regulated differentially by PIP_3_ concentration thresholds

The various PIP_3_-binding domains of signalling proteins bind 3′-phosphoinositides with different characteristic specificities and affinities. Some, such as the pleckstrin-homology (PH) domain of Bruton's tyrosine kinase (Btk) bind to PIP_3_ specifically and with high affinity [52]. The PH domains of the different isoforms of Akt bind to both PIP_3_ and PI(3,4)P_2_, but exhibit widely different affinities for those phospholipids [53]. Thus, the quality and quantities of PIP_3_-binding proteins recruited into a membrane-associated signal cascade will likely vary according to the concentrations of PIP_3_ generated in membranes by the stimulus. Phosphorylation of Akt requires the recruitment to membranes of Akt and two additional PIP_3_-binding proteins, PDK1 [54,55] and mSIN1 [56], which is a component of mechanistic target of rapamycin complex 2 (mTORC2). Because Akt phosphorylation requires three different proteins to bind to PIP_3_ near each other [57], Akt activation may require higher local concentrations of PIP_3_ than does recruitment of Btk or activation of PLCγ1. PI3K activity is stimulated by receptor-associated, PIP_3_-binding adapter proteins Gab1 and Gab2 [58,59]; their spatially organized activities could facilitate PIP_3_ concentration increases confined to cup domains of macropinosomes or phagosomes. Moreover, different PIP_3_ concentration thresholds within cups could define a sequence of PIP_3_-dependent effector activities associated with macropinosome formation.

### (e) Traffic of macropinosomes into lysosomes or back to the cell surface is regulated

Macropinosome closure creates an intracellular vacuole with enclosed water and solutes from outside the cell. The membrane bounding the nascent macropinosome differs from plasma membrane in several respects: Rab5a, PI3P and DAG are enriched in the membrane, whereas PI(4,5)P_2_ is depleted. The Rab5-positive macropinosome eventually matures to a Rab20/Rab21-positive organelle before acquiring Rab 7 [3,44,60,61]. Active Rab5a promotes the formation of tubulovesicular extensions [37], which are rich in PI3P-binding sorting nexins (Snx), especially Snx5 [62]. These morphological rearrangements alter the surface-to-volume ratio of the macropinosome vacuole and likely increase hydrostatic pressure inside the organelle [62]. Inhibition of Rab5 activity promotes macropinosome recycling to the plasma membrane [37]. Studies of PI3P dynamics during phagocytosis showed that concentrations of PI3P are integrated over phagosome membranes [63]. Thus, the concentrations of PI3P in macropinosome membranes and the attendant Rab5a activity could regulate whether macropinosomes recycle to the plasma membrane (low PI3P or Rab5 activity) or fuse with lysosomes (high PI3P or Rab5 activity).

Thus, macropinosome formation and maturation occurs by a regular sequence of cytoskeletal activities and changes in membrane chemistry. The networks of molecular interactions that organize these activities and the transitions from one activity to the next are likely to be affected by positive and negative feedback regulation.

## 3. Macropinocytosis-associated signals that modulate cell metabolism

To what extent are the chemical reactions organized to build the organelle also conveying other information that cells use? Ingestion of extracellular albumin by macropinocytosis and its subsequent digestion in lysosomes is sufficient to support the growth of K-Ras-transformed cells [13]. In this way, the macropinosome delivers a nutrient required for cell growth. Moreover, recent studies demonstrated that amino acids internalized by macropinocytosis can signal their presence in lysosomes [6] and that macropinocytic cups and macropinosomes localize essential elements of growth factor signalling which are unrelated to the process of macropinosome formation [64,65]. These studies suggest that macropinocytosis is more than an aberrant process of nutrient acquisition used by transformed cells, but is rather an essential component of signalling for normal cell growth.

### (a) Activation of mTORC1 requires ingestion of amino acids by macropinocytosis

The mechanistic target-of-rapamycin complex-1 (mTORC1) is a cytosolic protein complex that regulates metabolism in response to various inputs, including nutrient status and growth factor receptor signalling [66]. mTORC1 phosphorylates S6 kinase, 4EBP1 and other proteins which increase anabolic metabolism. Regulation of mTORC1 activity occurs on the cytosolic face of the lysosomal membrane. In nutrient-rich conditions, Rag GTPases associated with lysosomes recruit mTORC1 from cytosol onto those membranes, where mTORC1 can be activated by the GTPase Rheb [67,68]. The ability of Rag GTPases to recruit mTORC1 to lysosomes is regulated by a complex of lysosome-associated proteins called Ragulator, which integrates detection of amino acid concentrations in cytosol and lysosomes [69,70]. Elevated lumenal concentrations of the amino acid leucine in the lysosome signal to Ragulator through the lysosomal membrane proteins V-ATPase and SLC38A9 [71,72]. Ragulator then recruits mTORC1 from cytosol [73].

**Figure 4.**
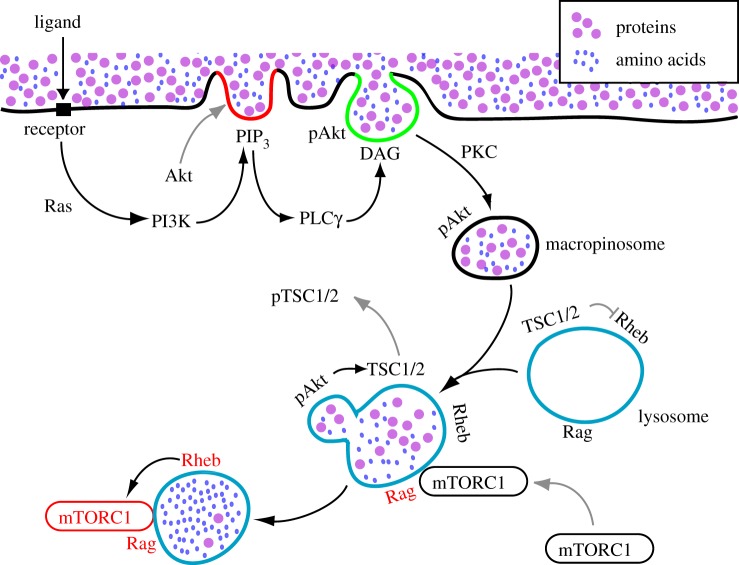
Receptor-mediated activation of mTORC1 by macropinocytosis of amino acids. Interaction between ligand and receptor activates Ras, PI3K and Rac, leading to membrane ruffling. Some ruffles change into cup-like structures, in which activated PI3K then transiently generates high local concentrations of PIP_3_ (red line). PIP_3_ in macropinocytic cups facilitates phosphorylation of Akt (pAkt) and the activation of PLCγ. pAkt is not required for macropinosome formation, but may be internalized on macropinosome membranes. PLCγ generates DAG in the cup (green line), leading to PKC-dependent pathways that close the macropinosome. Extracellular proteins or amino acids internalized by the macropinosomes are delivered rapidly into lysosomes through fusion reactions. Nutrient transfer from macropinosomes to lysosomes leads to activation of Rag GTPases (black to red), followed by mTORC1 recruitment from cytoplasm onto lysosomal membranes. pAkt generated in plasma membrane or macropinocytic cups may reach its substrate TSC1/2 via macropinosome traffic to lysosomes. Phosphorylation of TSC2 by pAkt leads to dissociation of pTSC1/2 from lysosome membrane, displacing a GAP activity that allows GTP loading of Rheb on lysosome membranes (black to red). GTP-bound Rheb activates mTORC1 on the lysosomal membranes (black to red).

Macropinocytosis provides a route for delivery of extracellular amino acids into lysosomes for the rapid activation of mTORC1. One biochemical model for measuring mTORC1 activation by growth factors or amino acids measures increased mTOR kinase activity following transient deprivation of growth factors or amino acids [74]. Re-addition of essential amino acids leads to activation of mTORC1 within 20 min in fibroblasts and HEK293 cells, which suggests that amino acids reach the lysosome lumen from outside the cell within that time period. Macropinocytosis can deliver solutes into lysosomes that quickly [6]. Growth factor stimulation enhances the activation of mTORC1 by amino acids. Moreover, activation of mTORC1 by amino acids also requires proteins that regulate endocytic membrane traffic, including Rab5a, Arf1 and enzymes which increase levels of the endolysosomal phospholipid PI(3,5)P_2_ [75–77]. Inhibition of macropinocytosis in macrophages and fibroblasts, by the sodium-proton antiporter inhibitor ethylisopropylamiloride, by the cytoskeletal inhibitors jasplakinolide and blebbistatin, or by depletion of Rac1, leads to decreased phosphorylation of S6K in response to leucine and growth factors [6]. Upregulation of macropinocytosis by acute growth factor stimulation increases the rate and efficiency of extracellular leucine delivery into lysosomes, permitting the recruitment and activation of mTORC1 on lysosomes (figure 4). This macropinocytosis-dependent pathway of mTORC1 activation serves as a vesicular mechanism of growth factor receptor signal transduction. Discovery of this dependency of mTORC1 activity on macropinocytosis led to the proposal that anabolic metabolism leading to cell growth is supported by amino acids internalized by macropinocytosis, or by similarly internalized proteins which are degraded to amino acids within lysosomes [6].

However, this concept of macropinocytosis-dependent cell growth was not supported by experimental models measuring cell proliferation instead of acute stimulation of mTORC1. Cells transformed by oncogenic Ras proteins exhibit increased macropinocytosis [78], which is required for protein synthesis and cell proliferation when albumin is provided as a source of amino acids [13,14]. By contrast, macropinocytosis is not required for proliferation of fibroblasts or Ras-transformed cells when amino acids are provided instead of albumin [79]. These studies suggest that amino acids reach mTORC1 and amino acid-detection mechanisms in cytosol by direct import of amino acids through transport proteins in the plasma membrane. The different results are not easily reconciled, especially for fibroblasts, which showed macropinocytosis-dependent activation of mTORC1 by amino acids [6] but macropinocytosis-independent proliferation in amino acids [79]. We speculate that the difference is due to mechanisms of cell growth or proliferation that are independent of mTORC1 activity [14,80]. Nonetheless, activation of mTORC1 by macropinocytosis-dependent delivery of leucine into lysosomes is clearly detectable in acute responses to stimulation. Whether other amino acids are capable of this macropinocytosis-mediated signalling to mTORC1 remains unknown.

### (b) The actin cytoskeleton differentially stimulates receptor-dependent phosphorylation of Akt

In addition to Rag GTPases, growth factor receptor signalling to mTORC1 requires that lysosome-associated Rheb be in its active, GTP-bound form. An essential component of Rheb activation is the kinase Akt, which is required for the activation of mTORC1 but not for macropinosome formation [34]. Rheb activity on lysosome membranes is maintained at low levels by the GTPase-activating protein (GAP) complex TSC1/2 [81], which also locates at the lysosomes. Growth factor receptor signalling activates Rheb via PI3K, which synthesizes PIP_3_ in membranes. The PIP_3_-rich membrane recruits Akt and the kinases PDK1 and mTORC2, which phosphorylate Akt [57]. Phospho-Akt phosphorylates TSC2, leading to the dissociation of TSC1/2 complex from lysosome membranes and separation from its substrate Rheb [82,83]. Thus, Rheb activity toward mTORC1 is increased by signalling that activates Akt to displace the Rheb GAP TSC1/2.

Whether macropinocytosis affects growth factor receptor signalling to Rheb via Akt remains largely unresolved. In our initial characterization of signalling in macrophages and fibroblasts stimulated by M-CSF and platelet-derived growth factor (PDGF), respectively, we observed that inhibition of macropinocytosis blocked S6K phosphorylation (a measure of mTORC1 activity) but did not reduce Akt phosphorylation [6]. This indicated the existence of a cytoskeleton-independent pathway from growth factor receptors to Rheb via PI3K, Akt and TSC1/2. However, a requirement of the actin cytoskeleton for growth factor-induced Akt phosphorylation has been demonstrated previously [84], and subsequent studies of macrophages stimulated with CXCL12 showed that cytoskeletal inhibition reduced phosphorylation of not only S6K, but also Akt and TSC2 [65]. Thus, CXCL12 initiates a cytoskeleton-dependent pathway leading to phosphorylation of Akt and TSC2. This suggests that ruffles or macropinocytic cups formed in response to CXCL12 amplify PI3K activity or otherwise locally stimulate phosphorylation of Akt.

Why is the actin cytoskeleton required for Akt phosphorylation in response to CXCL12 but not to M-CSF or PDGF? The signals generated by CXCR4, the G-protein-coupled receptor that binds CXCL12, may be qualitatively different from the signals generated by the M-CSF receptor, such that Akt phosphorylation occurs by distinct biochemical pathways which are differentially dependent on the actin cytoskeleton. However, we observed in macrophages that the maximal Akt phosphorylation response to CXCL12 was significantly lower than the maximal response to M-CSF [65]. We hypothesized that receptor signalling which generates low concentrations of PIP_3_ in membranes may be enhanced by cytoskeleton-dependent amplification of PI3K activity, such as occurs in macropinocytic cups. Consistent with this model, stimulation of macrophages with lower concentrations of M-CSF (140 pM), which generated lower levels of Akt phosphorylation than did receptor-saturating concentrations of M-CSF (6.9 nM), could be reduced by cytoskeleton inhibitors [65]. A similar relationship between the maximal levels of Akt phosphorylation responses and sensitivity to cytoskeletal inhibition was also observed in fibroblasts stimulated with PDGF, which generates a high maximal Akt response, and epidermal growth factor, which generates a low maximal Akt response [85]. These studies indicate that the actin cytoskeleton can amplify PI3K activity locally, and suggest that ruffles, macropinocytic cups or macropinosomes may serve as platforms for localized amplification of Akt phosphorylation by both PDK1 (generating pAkt(308)) and mTORC2 (pAkt(473)) [85]. Receptors with lower potential for generating PIP_3_ in membranes may be better suited to modulation of PIP_3_ concentrations by the actin cytoskeleton and therefore better suited for organizing cytoplasmic signalling spatially.

We proposed that macropinocytic cups or dorsal ruffles create large subdomains of the plasma membrane which localize signal amplification and propagation [86]. Such domains may restrict diffusion of enzymes, substrates and/or products, which could lead to locally elevated concentrations of PIP_3_. Alternatively or additionally, Akt phosphorylation may be directly increased by Rac1-dependent cytoskeletal activity. The Rac1 effector PAK, which is also required for macropinosome formation, can enhance Akt phosphorylation by serving as a scaffold for both Akt and PDK1 [87].

## 4. Possibilities

Whether macropinosomes form spontaneously or in response to receptor ligation, the spatially confined biochemical reactions leading to their formation are a kind of signal transduction. Macropinosomes are also capable of signalling to mTORC1 by delivering leucine or other amino acids into lysosomes, and macropinocytic cups provide platforms for localized activation of Akt. Additional roles for macropinocytosis in cell signalling, and for cell metabolism to feedback regulate signalling for macropinocytosis, are largely speculation. The distinct molecular constituents of macropinosome membranes may render them as platforms for signalling from internalized receptors [64,88]. The increased volume of ingested water and its subsequent flux across membranes and out of the cell could regulate cell volume or convey other information about extracellular conditions. Several additional possibilities are offered here.

### (a) Akt phosphorylated at the plasma membrane could reach its substrate TSC2 on lysosomal membranes via macropinocytosis

As described above, activation of PI3K by growth factor receptors leads to Akt phosphorylation in plasma membranes and eventually to Rheb activation on lysosomal membranes. The Rheb GAP TSC1/2 dissociates from lysosomal membranes when TSC2 is phosphorylated by phospho-Akt, thereby permitting activation of mTORC1 by Rheb [89]. It is not yet known how phospho-Akt at the plasma membrane accesses TSC2 on lysosomal membranes, but the enzyme and its substrate may be brought together via macropinocytosis (figure 4). Akt is present in macropinocytic cups [90] and has been localized to macropinosomes [64,65], where it may be en route to lysosomes.

### (b) Signalling in constant concentrations of growth factors may be stochastic and contingent on the formation of ruffles and cups

How important is macropinocytosis to receptor-regulated growth in metazoan cells? We propose that, in cells which are proliferating in constant concentrations of growth factor, receptor signalling is confined to cups and amplified by the cytoskeleton. As a mechanism to coordinate cell proliferation with organogenesis, growth factor-mediated regulation of cell growth may have co-opted macropinocytosis as an ancient mechanism of nutrient acquisition and feedback regulation. However, not all cells are as robustly macropinocytic as macrophages and tumor cells, and it remains to be determined whether macropinosomes form frequently enough or convey sufficiently strong signals to explain growth factor-dependent growth in other metazoan cells. Maybe when PI3K is active constitutively then other forms of endocytosis, less efficient than macropinocytosis, can deliver sufficient amino acids into lysosomes to activate mTORC1 and to support slow cell growth. Alternatively, amino acid transport proteins in plasma membrane of some cells may obviate the need for endocytic mechanisms of activating mTORC1. In that case, macropinocytosis may be unnecessary or function simply as a mechanism for clearing solutes and membranes.

### (c) Nutrient status or cellular dimensions may feedback regulate macropinocytosis

Cellular metabolic status can modulate macropinocytosis. Inhibition of mTORC1 or Akt increases rates of protein degradation in lysosomes without affecting rates of macropinosome formation [79], which suggests that nutritional status, signalling through mTORC1 or Akt, affects macropinosome-lysosome fusion. More generally, these observations suggest that starvation or nutrient sufficiency regulates the signalling pathways that initiate or complete macropinosome assembly or that mediate macropinosome fusion with lysosomes. As a mechanism for scavenging extracellular nutrients, macropinocytosis is likely to be regulated by cellular metabolic status, just as metabolic status regulates degradation of cytoplasm by autophagy [91].

The large-scale ingestion of membrane and extracellular fluid that occurs during macropinosome formation changes cellular dimensions by decreasing cell surface area and increasing cell volume. In earlier experiments, we examined the effects of cell dimensions on pinocytosis by asking whether macrophages recognize satiety. We reasoned that if cells can monitor their dimensions, then they should be capable of responding to the enlargement of their lysosomal compartment that follows phagocytosis. We determined that expansion of the lysosomal compartment by phagocytosis of a latex bead meal or by ingestion of sucrose did not affect rates of basal pinocytosis but significantly inhibited pinocytosis in macrophages stimulated by phorbol myristate acetate (PMA), which we later determined to be macropinocytosis [9]. Moreover, the more cells ate, the less they drank [92,93]. The mechanism by which expansion of the lysosomal compartment or overall cell size inhibits macropinocytosis remains unknown, but it indicates the existence of a feedback regulation of macropinocytosis by cellular dimensions. We speculate that PI3K, PTEN and PIP_3_ are components of a mechanism that translates cell dimensions into feedback regulation of cell behaviour, including macropinocytosis, phagocytosis and chemotaxis.

## 5. Conclusion

At the very least, macropinosomes organize and propagate signals necessary for their own construction. Their formation and maturation are subject to regulation by other inputs, and the distinct features of macropinosomes may influence other cell activities. The same signals that stimulate cell growth and oriented cell motility also direct macropinosome formation, which suggests that macropinocytosis itself is essential to cell growth. Although macropinosomes are capable of activating mTORC1 through delivery of amino acids into lysosomes, it is not yet clear whether these activities are required for cell growth. Macropinosomes may serve transient regulatory functions associated with their distinct compartmental identity within cytoplasm. That is, cells may be responsive to the abrupt appearance in cytoplasm of large acidic organelles with bounding membranes similar to plasma membrane but depleted of PI(4,5)P_2_ and rich in PI3P, which possibly also maintain a membrane electrical potential or high internal hydrostatic pressure. Moreover, cells may call on such organelles to deal with unusual situations, such as the appearance in cytoplasm of pathogens or other disruptive particles [94].

## Supplementary Material

Video 1

## Supplementary Material

Video 2

## Data accessibility

This article has no additional data.
